# Do Deterrence Mechanisms Reduce Cyberloafing When It Is an Observed Workplace Norm? A Moderated Mediation Model

**DOI:** 10.3390/ijerph18136751

**Published:** 2021-06-23

**Authors:** Mengmeng Song, Joseph Ugrin, Man Li, Jinnan Wu, Shanshan Guo, Wenpei Zhang

**Affiliations:** 1 Department of Business Administration, School of Business, Anhui University of Technology, Ma’anshan 243032, China; smmnzzx@163.com (M.S.); liman8607@126.com (M.L.); gssiris@163.com (S.G.); riverleafs@163.com (W.Z.); 2 Department of Accounting, College of Business Administration, University of Northern Iowa, 1227 West 27th Street, Cedar Falls, IA 50614, USA; joseph.ugrin@uni.edu

**Keywords:** cyberloafing, observability, perceived norms, certainty of sanctions, severity of sanctions

## Abstract

Despite the documented individual, job, and organizational antecedents of cyberloafing at the workplace, few studies have addressed whether, how and when group factors affect employees’ cyberloafing behaviors. Drawing on social learning theory and general deterrence theory, the purpose of this study is to test if observability of coworkers’ cyberloafing behavior affects employees’ perceptions of norms related to cyberloafing and subsequent cyberloafing behaviors and to test if sanctions can play a role in buffering these effects. An investigation of 335 employees working at Chinese enterprises establishes that observing others engaging in cyberloafing influences the employees’ perceived norms and cyberloafing behaviors and that employees’ perceived norms related to cyberloafing play a partial mediating role in the relationship between observability and employees’ cyberloafing. As predicted, we also found that perceived certainty and severity of potential sanctions for cyberloafing moderate the effect of observability on employees’ cyberloafing as well as the indirect effect of observability on employees’ cyberloafing via perceived norms related to cyberloafing. This study enriched the cyberloafing literature by revealing how observability of cyberloafing influences employees’ cyberloafing and by unveiling two boundary conditions under which the cyberloafing learning effect can be buffered.

## 1. Introduction

The Internet, mobile devices, and social media are widely used for work and pleasure and are entrenched elements of our daily lives [1,2]. The boundaries between work and non-work are increasingly blurring [3], and employees commonly engage in non-work online activities in the workplace. Restubog et al. [4] found that approximately 30–50% of employees use the Internet for non-work activities during the workday. Internet use at work for personal purposes is commonly called cyberloafing. Typical forms of cyberloafing include receiving or sending personal emails, browsing news, and shopping, amongst other things [5]. Studies show that employees’ cyberloafing can be destructive [6], lead to employees’ fatigue and a reduced focus on work, and in turn, cause a decline in productivity and output quality [7,8].

Employees’ cyberloafing has become a significant concern for managers and a hot topic for academic scholars due to the potential consequences [9]. Researchers have examined antecedents of employees’ cyberloafing in individual, job, and organizational contexts. Factors such as gender, education, personality, Internet experience, overqualification, and other individual characteristics influence employees’ cyberloafing [10,11,12,13,14,15]. Job-related factors such as stress, emotional conflict, burnout, overload, and boredom affect individuals’ propensity to cyberloaf [16,17,18,19]. Organizational factors which influence cyberloafing include organizational justice or injustice, organizational commitment, affective commitment, leader–member exchange, and punishment [5,19,20,21,22]. Unfortunately, however, group-level factors have been underestimated. Research shows that people’s behavior is easily affected by the behavior of coworkers and supervisors, as essential parts of the group, which are similar to or close to themselves [23,24]. As behaviors become widespread, they become group norms, and norms influence more future behavior. Thus, it is important to explore how employees respond to norms related to workplace cyberloafing behaviors that they learn from observing coworkers and supervisors.

Two recent studies show the relationship between coworkers’ cyberloafing and employees’ cyberloafing [25,26]. However, such studies on the interpersonal effect of cyberloafing suffer from several shortcomings. First, research has not examined the underlying mechanisms through which coworkers’ cyberloafing influences employees’ cyberloafing. We propose that perceived norms developed through observing others’ cyberloafing influences employees’ cyberloafing and mediates the relationship between observability and cyberloafing behavior. The mediating effect is based on social learning theory [27,28] which suggests employees learn from observing others, resulting in norms. Second, despite the significant effect of observability on employees’ cyberloafing, the current literature offers little insight into moderators that can weaken this effect. To fill this research gap, this study identifies two conditions, perceived certainty and severity of sanctions, which have been found to reduce cyberloafing [29,30,31], and tests their moderating effect on the relationship between observability, perceived norms, and employees’ cyberloafing behavior. We base our propositions on Beccaria’s general deterrence theory (GDT) [32]. Finally, prior research focused on individuals in the U.S., an individualistic culture. It is unclear whether research findings are robust in the context of a collectivistic culture. Given that individuals in collectivist countries are more susceptible to peoples’ influence to achieve internal harmony [28], it is important to update existing findings with evidence from collectivist countries (e.g., China in this study).

The remainder of the study describes the theoretical foundation underlying this study and the model, hypotheses proposed, the research methodology, and the results, and finally makes a final discussion and conclusion.

### 1.1. Theoretical Foundation and Research Model

The social learning theory argues that individuals learn about group norms and acceptable behaviors by observing others [27,28]. Specifically, employees’ behaviors are often similar to coworkers, because they spend more time with coworkers than other strangers and are more likely to be influenced by them [33]. Therefore, for employees, the behavior of coworkers has a certain reference significance. Akers, et al. [34] suggested that individuals determine whether to learn others’ deviant behavior by judging whether this behavior is acceptable or not. If some deviant behavior (e.g., cyberloafing in this study) is found to be tolerant by most individuals, it can develop into a group norm which makes individuals more likely to imitate them [27,34,35,36]. The social learning process of this kind of deviant behavior also exists in cyberloafing at workplace.

Social learning theory also points out that the individual’s perception of reinforcement strengthens or weakens the individual’s learning behavior [28,34]. Reinforcement is divided into positive reinforcement and negative reinforcement. As a negative reinforcement measure, sanctions can weaken the individual’s social learning behavior to a certain extent [37]. In order to avoid sanctions, the individual reduces the possibility of learning deviant behavior. The deterrence theory divides sanctions into the certainty of sanctions, the severity of sanction, and the celerity of sanctions. However, more studies have established that the certainty and severity of sanctions have a certain effect on the deviant behavior of employees [30,38]. Therefore, our study further explores the inhibitory effect of individual perception of certainty and severity of sanctions on the process of employees’ behavior learning of cyberloafing.

**Figure 1:**
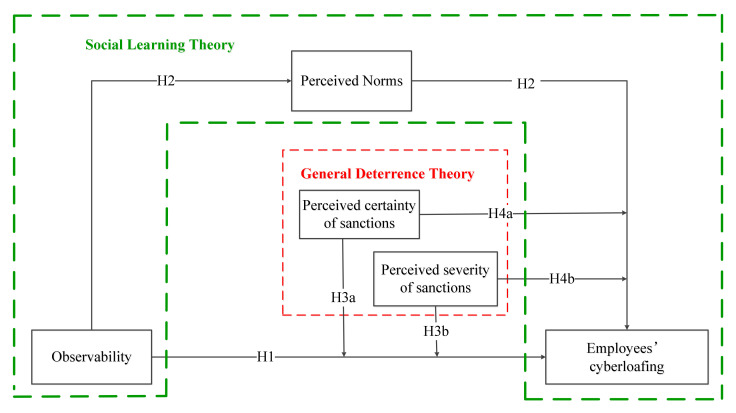
Research model.

This study draws on social learning theory and general deterrence theory to theoretically established an integrated framework, which is more suitable for answering three related research questions: (1) Whether observability influences employees’ cyberloafing (H1)? (2) How observability influences employees’ cyberloafing (H2)? (3) When observability influences employees’ cyberloafing (H3a, H3b, H4a, and H4b)? Thus, the integrated research model displayed in Figure 1 is proposed to reveal the direct effect of observability on cyberloafing, the mediating effect of perceived norms on the observability-cyberloafing link, and the moderating effect of perceived certainty and severity of sanctions on the observability-cyberloafing link and on the perceived norms-cyberloafing link.

### 1.2. The Main Effect of Observability and Employees’ Cyberloafing

Cyberloafing can be considered a deviant workplace behavior if it breaks rules and ultimately wastes time and resources, and damages the organization [39,40]. Deviant workplace behavior is commonplace [41], and cyberloafing is particularly problematic as Restubog, Garcia, Toledano, Amarnani, Tolentino, and Tang [4] found that approximately 30–50 percent of employees use the Internet for non-work activities during the workday, and the data show that more than half of the time employees use the Internet during the workday is non-work related [42]. Other data also show that over 60 percent of organizations have reprimanded and over 30 percent have terminated employees for cyberloafing [43].

Social learning theory states that people learn how to engage in their environment by learning through social exchange and social relationships [28]. The workplace is made up of a complex network of social exchanges and influences that impact how employees feel and behave. Employees observe, learn, and react to the complex workplace environment. Robinson and O’Leary-Kelly [27] state that “individuals carefully analyze their work environments and adjust their individual actions accordingly.” Akinyele [44] find that workplace productivity problems can namely be attributed to the work environment and cyberloafing behavior is affected by a wide range of workplace factors, including employees stress [45], mechanisms for self-management [46], Internet monitoring [9], and the use of policies and sanctions [14], amongst other things.

The workplace also facilitates social exchange, including through generalized exchange [47], which is an indirect exchange between group members where they receive feedback indirectly through observation [48]. Thus, the workplace is greatly affected by social learning. Sutherland [49] introduced differential association theory to argue that individuals learn social behavior through interaction with intimate others and that through interactions, people develop attitudes, perceptions, values, motives, and ultimately behaviors. Sutherland asserts that people are influenced by both the frequency and intensity of their interactions with others. Bandura [28], Burgess and Akers [50], and Akers [37] connect differential association theory with social learning theory that conceptualizes the interplay between differential association, imitation, and differential reinforcement.

Differential association [49] relates to how people formulate decisions to engage in deviant behavior by learning about others’ behaviors through personal interactions. Bandura [28] and Akers [37] further assert that indirect interaction and indirect information influence imitation. Both Akers [37] and Sutherland [49] suggest that the ultimate effects are influenced by the duration of the interaction. Imitation is the byproduct of social learning [51], and researchers have already shown that employees imitate other employees’ behaviors such as absenteeism [52]. Considering social learning theory and evidence that the workplace facilitates social learning and employee imitation of one another, we hypothesize that when employees observe coworkers cyberloafing, they are more likely to cyberloaf themselves.

**Hypothesis** **1** **(H1).** *Observability is positively associated with employees’ workplace cyberloafing*.

### 1.3. The Mediating Effects of Perceived Norms on the Relationship between Observability and Employees’ Cyberloafing

Bandura’s [28] social learning theory explains how individuals amass information about their social environment and how to behave. Individuals emulate role models and follow the standardized norms of the environment. Robinson and O’Leary-Kelly [27] suggest that worker behaviors set the standards for normative and acceptable behavior, which employees emulate. From that perspective, workplace cyberloafing is a learned behavior where employees are influenced by standardized workplace norms, including norms related to cyberloafing that are learned through observation. Thus, greater observability leads to stronger perceptions that workplace cyberloafing is a normal behavior and vice versa.

Social learning theory says people seek ways to justify improper behaviors [53]. One way to justify behavior is to have consistent group norms, which can be learned through interaction with others in the environment. We see this phenomena in other contexts such as software piracy where people believe engaging in unethical or even illegal behavior is acceptable if most people do it, and the more people that do it, the firmer the belief [54]. Bandura [28] and Willison [55] argued that norms and the “everyone else does it” excuse for self-justification can be learned through the social environment. This view is supported by Blanchard and Henle [56], who propose that employees do not consider cyberloafing during working hours to be inappropriate because their colleagues or supervisors do the same. Lim and Teo [57] find that “About 88% of respondents reported that it is acceptable to use company Internet access to cyberloaf when they perceived that everyone else engaged in it.” Along the same vein, Askew, et al. [58] shows that peoples’ perceptions of norms related to cyberloafing affect their attitude, and attitudes are the precursor to behavior. Thus, social learning theory supports our conjecture that observing others’ cyberloafing in the workplace influences perceived norms, which influence cyberloafing behavior. Increased observability creates an opportunity for social learning and the development of norms, which in turn influence behavior as a form of neutralizing. Thus, we hypothesize:

**Hypothesis** **2** **(H2).** *Perceived norms as related to cyberloafing mediate the relationship between observability and employees’ workplace cyberloafing*.

### 1.4. The Moderating Effect of Perceived Certainty and Severity of Sanctions on the Relationship between Observability and Employees’ Cyberloafing

General deterrence theory (GDT) [32] is a criminal justice theory that has been used to examine the effects of sanctions and consequences on cyberloafing [14]. GDT proposes that policies and regulations, imposed on individuals by authorities (such as organizations), affect individual attitudes, choices, and actions. The key premise behind this model is that individuals make rational decisions to benefit themselves. GDT assumes individuals weigh potential consequences for taking an action [14,32,59]. Furthermore, GDT is more effective at deterring behaviors that are engaged in by rational choice [60].

GDT suggests that perceived certainty and severity of sanctions are the two most effective deterrence mechanisms [61]. In this paper, perceived certainty of sanctions refers to the possibility that employees are caught when they engage in cyberloafing, and perceived severity of sanctions is defined as the perception that severe sanctions take place if caught. Previous studies have shown that when individuals are aware of the high visibility of misbehavior, sanctions imposed by the working group may weaken the spread of organizational misbehavior to individuals [24], employees reduce the occurrence of the behavior which is more likely to be detected, and the potential negative consequences for this behavior are likely to be severe [32,62,63]. Robinson and O’Leary Kelly found that there is a positive relationship between the anti-social behavior of individuals and the degree of anti-social behavior of working group members, and the management sanctions for this behavior moderates the relationship between them [27]. Berry and Westfall pointed out that more than 60% of college students said they were less likely to use their mobile phones in class if they saw their classmate punished (for example, confiscating their phone or demoting) [64]. Similarly, Brinda and Basu [65] links immediate sanctions with significantly reduced employees’ cyberloafing once the cyberloafing was detected. To put it differently, perceived certainty and severity of sanctions in the condition of high perception could effectively reduce employees’ cyberloafing. Thus, we hypothesize:

**Hypothesis** **3a** **(H3a).** *Perceived certainty of sanctions moderates the association between observability and employees’ workplace cyberloafing*.

**Hypothesis** **3b** **(H3b).** *Perceived severity of sanctions moderates the association between observability and employees’ workplace cyberloafing*.

### 1.5. The Moderating Effect of Perceived Certainty and Severity of Sanctions on the Relationship between Perceived Norms and Employees’ Cyberloafing

We predict that perceived norms about workplace cyberloafing are augmented by externally imposed sanctions and consequences for such behavior. Group norms are not only norms that support employees’ cyberloafing, but they can be transformed by sanctions to reflect that cyberloafing is either not admitted or tolerated. Strong sanctions punishing employees for workplace cyberloafing mitigate the effects of beliefs that cyberloafing is a commonly accepted practice, whereas weak sanctions amply normative beliefs that cyberloafing is acceptable.

Consistent with the central tenets of GDT, D’Arcy, Hovav, and Galletta [30] found that certain sanctions can reduce cyberloafing, and Ugrin and Pearson [14] found that employee cyberloafing can be deterred when employees perceive potential sanctions to be severe and enforced. In a recent field study, Hensel and Agnieszka [22] find that the perceived certainty and severity of sanctions send a strong signal, and they conclude that the perception that consequences are likely to be certain and severe improve people’s understanding and awareness of the view that the behavior is wrong, and perceptions of wrongdoing reduce illicit behavior [38,66].

In addition to deterring behavior directly, we propose that perceptions about potential consequences interact with observations of workplace behaviors that develop into workplace norms. Individuals make further decisions based on contact with information that supports or conflicts with the behavior [67]. When the organization conveys information about sanctions to employees, employees may change their previous attitudes or decisions if the sanctions are severe; even if this behavior conforms to the group norms, individuals still reduce or suspend cyberloafing. For example, Lee and Lee [68] found that individuals are less likely to cyberloaf in the workplace when they have observed individuals get punished for such behavior. However, weak sanctions reinforce previous attitudes or decisions. Friedman, Simon, and Liu pointed out that when individuals observes unpunished organizational misbehavior, they are more likely to hold the view that this behavior is normative and are more likely to engage in similar behaviors [69]. Observations of coworkers engaging in cyberloafing may create a normative workplace behavior, as hypothesized above, while observations of sanctions being levied against individuals that cyberloafing should effectively moderate any relationship between perceptions of norms and workplace cyberloafing. Considering the aforementioned evidence, it is reasonable to conclude that even when individuals’ perceptions that cyberloafing is a normal behavior in the workplace, fear about the certainty and severity of sanctions for engaging in cyberloafing should moderate any relationship between perceived norms and workplace cyberloafing. Thus, we hypothesize:

**Hypothesis** **4a** **(H4a).** *Perceived certainty of sanctions moderates the association between perceived norms and employees’ workplace cyberloafing*.

**Hypothesis** **4b** **(H4b).** *Perceived severity of sanctions moderates the association between perceived norms and employees’ workplace cyberloafing*.

## 2. Materials and Methods 

### 2.1. Sampling and Procedures

We tested our model using an online survey to collect self-reported data for testing the hypotheses. To ensure the representativeness of the sample, we targeted at full-time employees working in enterprises located in the Internet, catering and tourism, manufacturing, finance, and real estate industries, in 25 different provinces in central and eastern China (e.g., Guangdong, Shanghai, Jiangsu, Hebei, and Beijing). This study was approved by the Ethics Committee of Anhui University of Technology (YXLLSP20201202 and 20.05.2020). All participants gave their informed consent for inclusion prior to the survey. In order to decrease potential effect of socially desirable responding on data quality, our questionnaires were distributed to participants on an online survey platform (www.wjx.com, accessed date: 20 June 2021), which has collected 7.119 billion responses for its users, to keep high levels of anonymity and more reliability in collecting sensitive information [70,71]. Specifically, with the paid service from (www.wjx.com, accessed date: 20 June 2021) researchers first asked it to send questionnaires to Internet users of enterprises in a variety of designated industries. Then, the questionnaires were randomly sent to target participants with a quick response (QR) code through WeChat, one of the most popular social media application in China and all over the world [72].

The data collection lasted for 3 weeks, from 20 May to 10 June 2020, and participants could access questionnaire from computers and mobile devices during off-duty hours (i.e., 18:00–23:00). Before filling the questionnaires, participants were asked to take about 3 min to understand the purpose and instructions of the survey, and then it took them about 2 min to complete all the questionnaires. Each participant received a reward (e.g., member points in the survey platform) worth about 2 RMB (approximately equal to 30 cents) after completing the questionnaires. Furthermore, followed by previous studies like Wu, Mei, Liu and Ugrin [8], we set IP address recognition to ensure that the questionnaire can only be filled out once with the same IP and set a strict time limit that if it took less than 2 min or more than 10 min, it would be considered as an invalid questionnaire. In total, we randomly distributed 397 questionnaires in our online survey. After eliminating invalid and incomplete responses, a total of 335 valid questionnaires were obtained, with an effective response rate of 84.38%.

Of the 335 responses, 59.7% of them were female, 62.1% of them were married, and 83.0% of them worked in lower-level management positions or below. The participants were evenly distributed among different income groups: less than 3000 Yuan (11%), between 3001 and 5000 Yuan (18.2%), between 5001 and 7000 Yuan (29.9%), between 7001 and 10,000 Yuan (22.7%), and more than 10,000 Yuan (18.2%). The most common educational level was university or junior college (85.6%), followed by master’s or above (9.9%), senior or technical secondary school (3.9%), and junior high school or below (0.6%). Working years included less than 3 years (19.1%), between 3 and 5 years (20.9%), between 6 and 7 years (26.0%), between 8 and 10 years (13.7%), and more than 10 years (20.3%). 

### 2.2. Measures 

We adapted several scales that have been validated in other research for use in our survey instrument. All items were scored with 7-point Likert scales. A summary of the scales are as follows:

Observability (OS): We measured observability by adopting the two-item scale created by Siponen, et al. [61]. The items state, “In my organization, employees’ use of the Internet at work for non-work-related activities is widely visible” and “In my organization, employees’ use of the Internet at work for non-work-related activities is visible in public.” The Cronbach’s alpha for our responses was 0.892, indicating the items are reliable.

Perceived Norms (PN): We measured perceived norms by adapting the four-item scale created by [54]. The items are as follows: “If it were prevalent in the company to use the Internet at work for non-work-related activities, and if a lot of people were doing it?”, “If it were held that other people are benefiting from using the Internet at work for non-work-related activities, and why should not I?”, “If it were held that no one else seems to care whether or not they get caught when they use the Internet at work for non-work-related activities?”, and “If using the Internet at work for non-work-related activities makes me feel at least a little more ‘cool’.” The Cronbach’s alpha for the four-item scale was 0.799, indicating the scale is reliable.

Perceived certainty of sanctions (CS): We assessed perceived certainty of sanctions by adapting a three-item scale created by Siponen and Vance [61]. The items are as follows “What is the chance you would receive sanctions if you engage in cyberloafing during working hours?”, “What is the chance that you would be formally sanctioned if management learned you had used the Internet at work for non-work-related activities?”, and “What is the chance that you would be formally reprimanded if management learned you had used the Internet at work for non-work-related activities?” The Cronbach’s alpha was 0.843, indicating the scale is reliable.

Perceived severity of sanctions (SS): We assessed perceived severity of sanctions by adapting a three-item scale created by Siponen and Vance [61]. The items are as follows: “How much of a problem would it create in your life if you were formally sanctions for cyberloafing during work hours?”, “How much of a problem would it create in your life if you were formally sanctioned for using the Internet at work for non-work-related activities?”, and “How much of a problem would it create in your life if you were formally reprimanded for using the Internet at work for non-work-related activities?” The Cronbach’s alpha was 0.811, indicating the scale is reliable.

Employees’ cyberloafing (EC): We measured employee cyberloafing using the three-item scale adopted from Moody and Siponen [73]. The items are as follows: “In general, I use the Internet at work for non-work-related purposes.”, “I access the Internet at work for non-work-related purposes several times each day”, and “I spend a significant amount of time on the Internet at work for non-work-related purposes.” The Cronbach’s alpha was 0.818, indicating the scale is reliable.

Control variables: We included control variables that have correlated with employees’ cyberloafing in previous studies (see discussions in [5,8,74,75]). We control for gender (GD), education (ED), income (IC), marital status (MS), position (PS), and work experience (WY).

## 3. Results

### 3.1. Confirmatory Factor Analysis

To verify the convergent validity and discriminant validity of the scale, we analyzed the 335 questionnaires using Mplus 7.0 software (University of California, Los Angeles, USA). Table 1 displays the results of a confirmatory factor analysis of the measurement model. The model fit indices (χ^2^/df = 2.061, CFI = 0.957, TLI = 0.943, SRMR = 0.041, RMSEA = 0.056) suggest that the model is acceptable [76]. The standardized loadings for the variables range from 0.556~0.914, with all being over 0.5. The average of the variance extracted (AVE) of the latent variables ranged from 0.507 to 0.813, larger than the threshold of 0.50. The composite reliabilities (CR) ranged from 0.802~0.897, with all being higher than the recommended threshold value of 0.7, presenting good convergent validity for the scales. 

As suggested by Fornell and Larcker [77], the square root of AVE, by comparison, is greater than the correlation coefficient between other potential variables and this latent variable (Table 2), indicating that the discriminant validity among the five main variables in this study is good.

### 3.2. Common Method Biases Analysis

We tested for common method bias, which could have been introduced by the survey instrument, for all variables and consistent with Harman’s one-factor test [78] and a confirmatory factor analysis [79]. The principal axis factoring analysis was used to extract common factors and generated five principal components, accounting for 74.39% of the variance. The first principal component explains 30.20% of the variance. We also used robust maximum likelihood method to perform a confirmatory factor analysis and compared the fit indices of five competing models. The fit indices of the five-factor model (χ^2^/df = 2.061, CFI = 0.957, TLI = 0.943, SRMR = 0.041, RMSEA = 0.056) was considerably better (Δχ^2^ = 1200.4, Δdf = 10, *p* < 0.001) than that of the single-factor model (χ^2^/df = 15.169, CFI = 0.349, TLI = 0.241, SRMR = 0.174, RMSEA = 0.206) and other alternative models. Therefore, we concluded that there is little threat of common method bias in the data.

### 3.3. Correlation Analysis

Table 2 presents the means, standard deviations, and correlation coefficients for the variables assessed in the study. There is significant positive correlation between observability and perceived norms (*r* = 0.568, *p* < 0.01) and employees’ cyberloafing (*r* = 0.387, *p*< 0.01). There is also a positive correlation between perceived norms and employees’ cyberloafing (*r* = 0.308, *p* < 0.01). Employees cyberloafing has a significantly negative correlation with both perceived certainty (*r* = −0.216, *p* < 0.01) and severity of sanctions (*r* = −0.247, *p* < 0.01). Taken as a whole, the correlation analysis is consistent with the theoretical expectations, which lays a foundation for later hypothesis testing.

### 3.4. Mediation Effect Analysis

We tested for mediation using the procedure outlined in Zhao, et al. [80]. In doing so, we performed a Bootstrap analysis applying the SPSS PROCESS script developed by Hayes [81] and generated 5000 bootstrapped samples to test the mediating effect of perceived norms. The results are shown in Table 3. The relationships between observability, perceived norms, and employees’ cyberloafing are modeled using regression analysis. The results show that observability positively associates with employees’ cyberloafing and perceived norms. Furthermore, perceived norms are positively associated with employees’ cyberloafing. The bias-corrected bootstrap method shows that the 95% confidence interval for the path mediated by perceived norms is (0.003, 0.133), excluding zero, indicating that perceived norms partially mediates the relationship between observability and employees’ cyberloafing. Thus, H1 and H2 are supported.

### 3.5. Moderated Mediation Effect Analysis

**Figure 2:**
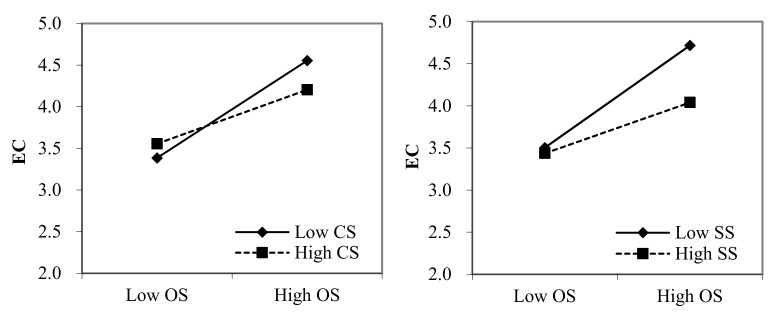
The interaction effect of OS and CS, as well as SS, on EC. Note: OS, observability; CS, perceived certainty of sanctions; SS, perceived severity of sanctions; and EC, employees’ cyberloafing.

**Figure 3:**
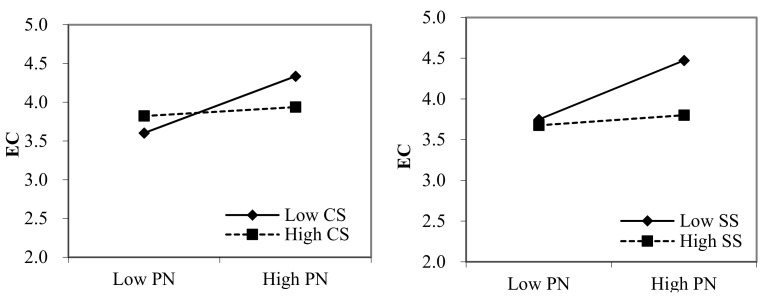
The interaction effect of PN and CS, as well as SS, on EC. Note: PN, perceived norms; CS, perceived certainty of sanctions; SS, perceived severity of sanctions; and EC, employees’ cyberloafing.

We estimated the moderating effect of perceived certainty and severity of sanctions on the relation between observability and employees’ cyberloafing and the relation between perceived norms and employees’ cyberloafing using the PROCESS macro (Model 17), as recommended by Hayes [82]. Table 4 illustrates that both the interactions between observability and perceived certainty, as well as the severity of sanctions, are negatively correlated with employees’ cyberloafing. This further suggests that perceived certainty and severity of sanctions negatively moderate the relationship between observability and employees’ cyberloafing. Thus, H3a and H3b are supported. Furthermore, the results show that the effect of perceived norms on employees’ cyberloafing is also moderated by perceived certainty and the severity of sanctions, supporting H4a and H4b. To further interpret these interaction effects, we plotted the two-way interactions. As shown in Figure 2, the relationship between observability and cyberloafing was stronger among employees who perceived that the certainty or severity of sanctions was low. Similarly, perceived norms have greater effect on cyberloafing when employees perceived low certainty or severity of sanctions (see Figure 3).

Index indicators were employed to further verify the moderated mediation effect in accordance with the approach suggested by Hayes [82]. Table 5 shows that the association between perceived norms and employees’ cyberloafing is stronger when perceived certainty and severity of sanctions are low but not significant under conditions of the other three combinations. The indexes of partial moderated mediation indicate that the mediation of perceived norms were moderated by both perceived certainty and severity of sanctions, because the 95% confidence interval was ranging from −0.107 to −0.0003 and from −0.134 to −0.011, respectively, not including zero.

## 4. Discussion

In recent years, increasing academic attention has been paid to antecedents of cyberloafing behavior at workplace because of its destructive effects on both organizations and employees. To deepen the understanding of what induces employees’ cyberloafing, the purpose of this study is to examine the effect of observability of cyberloafing on employees’ cyberloafing with perceived norms as a mediator and perceived certainty and severity of sanctions as two moderators from the perspective of social learning and deterrence. The findings show that observability of cyberloafing is positively associated with employees’ perceived norms and subsequent cyberloafing, and perceived norms partially mediate the relationship between observability and employee cyberloafing. This means that observability indirectly affects employees’ cyberloafing by increasing the likelihood that employees believe that cyberloafing is a workplace norm. The results also indicate that the perceived certainty and severity of sanctions buffer the relationship between observability and employee cyberloafing and alleviate the mediating effect of perceived norms. These findings suggest that when employees perceive that potential sanctions for cyberloafing are certain or severe, they are less likely to engage in cyberloafing activities when they observe others’ cyberloafing at workplace.

This study has several theoretical implications for cyberloafing research. First, it contributes to our understanding of cyberloafing by identifying a new antecedent of cyberloafing behaviors. Prior studies have found that cyberloafing is predicted by individual [4,83,84,85], job-related [86,87,88,89,90], and organizational factors [5,19,20,21] but neglected the potential effect of antecedents at group context. This study extends the scope of the extant research by exploring cyberloafing from the perspective of interpersonal social learning and reveals that observability of cyberloafing at workplace can be one of the reasons for understanding employees’ cyberloafing. Second, our results advance our understanding of social contagion of cyberloafing by examining the mechanism through which such behavioral contagion occurs. Although recent studies have provided preliminary evidence that coworkers’ cyberloafing is associated with employees’ cyberloafing in the U.S. with individualistic culture [25,26], the potential underlying mechanisms through which coworkers’ cyberloafing influences employees’ cyberloafing has not been discussed. This study thus provides further evidence from China, which has a significant collectivistic culture, to support these studies and expands existing findings by including perceived norms related to cyberloafing as a new mechanism underlying the influence of observability on employee cyberloafing based on social learning theory [28]. Third, this research contributes largely to the existing body of knowledge by filling the cyberloafing literature gap on a better understanding of two conditions under which observability influences employees’ cyberloafing behaviors, with a particular emphasis on the moderating role played by employees’ perception of sanction certainty and severity in predicting their response to others’ cyberloafing at workplace. Our findings demonstrate that a high level of perceived certainty or severity of sanctions can serve as an effective deterrence strategy for preventing social contagion of cyberloafing at workplace, thus broadening our knowledge on organizational situations inducing or resisting employees’ cyberloafing which have been examined in recent studies [15,91].

This study has important implications for management. First, management can expect that employee cyberloafing swells once it starts and if there are no negative consequences. As employees see others spending time online, they may be swayed to do so themselves. This may be exacerbated in modern open office environments. It seems that certain and severe consequences are more effectual in office layouts where peoples’ activities are observable. The management should reduce the screen visibility of employees by setting up compartments so that they cannot see what their coworkers are doing on the computer, thus reducing the possibility of cyberloafing learning. Second, employees who observe others around them engaging in cyberloafing often do not perceive cyberloafing to be a deviant behavior [56] and have little awareness of the negative effects of cyberloafing [92]. Therefore, strengthening the education of employees and advanced warning of the detrimental effects of cyberloafing may be necessary for enterprises to stop employees. Third, the results suggest employers, particularly in China, need to set clear rules through Internet-use policies and clearly define the consequences for cyberloafing [93,94]. Once policies are designed, consequences must be effectively put into practice when people violate rules, to be a warning that such behavior in the enterprise is inappropriate.

This study takes place in China, a country with a highly collective culture that may be more susceptible to influence from observations and norms. It would be interesting to see if these relationships hold true in a more individualistic culture, such as the United States. The study also has a relatively small sample, and it mainly comes from surveys of employees working at organizations located in the Yangtze River Delta, Pearl River Delta, and Bohai Bay. Thus, the sample may not even capture a holistic view of China, as China itself has many regional cultures. The study is also limited in that it only examines two facets of deterrence: certainty and severity. We test the effects of the perceived certainty and severity of sanctions on the relations between observability and perceived norms on employees’ cyberloafing, but we did not test the effects of the perceived celerity of sanctions or how quickly the punishment occurs [95]. Future research could explore this additional factor.

## 5. Conclusions

A growing body of studies on cyberloafing at workplace has established the negative effects of cyberloafing on employees’ job outcomes and mental health, and identified factors that predict employees’ cyberloafing behaviors. However, very few studies have examined how and when observability of cyberloafing at workplace influences employees’ levels of cyberloafing. The present study is the first study to investigate the effects of observability on employees’ cyberloafing with the mediation of perceived norms and test the moderations of perceived certainty and severity of sanctions in a sample of 335 employees in China. We substantiate that observability of cyberloafing decreases employees’ cyberloafing behaviors directly and indirectly by the enhancement of perceived norms. Furthermore, perceived certainty and severity of potential sanctions for cyberloafing moderate the effect of observability on employees’ cyberloafing and the indirect effect of perceived normalcy on it. Our results suggest that managers benefit from administering certain and severe sanctions on cyberloafing activities to avoid the ubiquitous office culture of cyberloafing.

## Figures and Tables

**Table 1:** Fit indices of the factor models.

Model	χ^2^	df	χ^2^/df	CFI	TLI	SRMR	RMSEA	∆χ^2^(∆df)
Single-factor model	1365.244	90	15.169	0.349	0.241	0.174	0.206	1200.4 (10) ***
Two-factor model	1104.685	89	112.412	0.482	0.389	0.169	0.185	939.9(9) ***
Three-factor model	900.956	87	10.356	0.585	0.499	0.169	0.167	736.1 (7) ***
Four-factor model	321.003	84	3.821	0.879	0.849	0.060	0.092	156.2(4) ***
Five-factor model	164.808	80	2.061	0.957	0.943	0.041	0.056	

Notes: OS, observability; PN, perceived norms; CS, perceived certainty of sanctions; SS, perceived severity of sanctions; EC, employees’ cyberloafing; single-factor model: OS + PN + CS + SS + EC; two-factor model: OS + PN + CS + SS, EC; three-factor model: OS + CS + SS, PN, EC; four-factor model: OS, PN, CS + SS, EC; and five-factor model: OS, PN, CS, SS, EC. *** *p* < 0.001.

**Table 2:** Means, standard deviations, and correlation coefficients of all variables.

Variables	Mean	SD	OS	PN	CS	SS	EC
OS	4.221	1.266	**0.902**				
PN	3.908	0.901	0.568 ***	**0.712**			
CS	4.228	1.211	−0.148 **	−0.090	**0.801**		
SS	4.895	0.977	−0.034	−0.080	0.506 ***	**0.769**	
EC	3.309	1.174	0.387 ***	0.308 ***	−0.216 ***	−0.247 ***	**0.780**

Note: OS, observability; PN, perceived norms; CS, perceived certainty of sanctions; SS, perceived severity of sanctions; and EC, employees’ cyberloafing. The square roots of AVE values are bold and reported in diagonal. ** *p* < 0.01, and *** *p* < 0.001.

**Table 3:** Multiple regression analyses of the mediation effect.

Variables	EC		PN		EC	
	β	t	β	t	β	T
GD	−0.094	−0.761	0.024	0.276	−0.099	−0.798
ED	−0.118	−0.750	0.040	0.367	−0.125	−0.797
IC	0.064	1.112	−0.019	−0.482	0.067	1.175
MS	−0.283	−1.625	0.071	0.591	−0.295	−1.703
WY	0.129	2.077	−0.004	−0.085	0.1298 *	2.098
PS	−0.141	−1.627	−0.056	−0.934	−0.131	−1.522
OS	0.368 ***	7.823	0.402 ***	12.349	0.299 ***	5.279
PN					0.172 *	2.160
R^2^	0.170		0.326		0.182	
F	9.592 ***		22.589 ***		9.070 ***	

Notes: OS, observability; PN, perceived norms; CS, perceived certainty of sanctions; SS, perceived severity of sanctions; and EC, employees’ cyberloafing; * *p* < 0.05, *** *p* < 0.001.

**Table 4:** The moderating effect of CS and SS.

	PN		EC	
	β	T	β	t
GD	0.024	0.276	0.025	0.230
ED	0.040	0.367	−0.196	−1.418
IC	−0.019	−0.482	0.017	0.337
MS	0.071	0.591	−0.199	−1.313
WY	−0.004	−0.085	0.095	1.731
PS	−0.056	−0.934	−0.069	−0.907
OS	0.402 ***	12.349	0.359 ***	6.989
CS			−0.037	−0.715
OS*CS			−0.085 *	−2.008
SS			−0.190 **	−3.037
OS*SS			−0.123 *	−2.094
PN			0.236 *	3.327
PN*CS			−0.142 *	−2.174
PN*SS			−0.171 *	−1.990
R^2^	0.326		0.390	
F	22.589 ***		14.589 ***	

Notes: OS, observability; PN, perceived norms; CS, perceived certainty of sanctions; SS, perceived severity of sanctions; and EC, employees’ cyberloafing; * *p* < 0.05, ** *p* < 0.01, *** *p* < 0.001.

**Table 5:** The moderated mediation effect of CS and SS.

Moderator Variable	Conditional indirect effects			
	Effect	Boot SE	95% CI	
			LLCI	ULCI
Low CS, Low SS	0.231	0.046	0.143	0.326
Low CS, High SS	0.097	0.058	−0.030	0.200
High CS, Low SS	0.093	0.062	−0.017	0.227
High CS, High SS	−0.041	0.043	−0.122	0.046
	Indices of partial moderated mediation			
	Index	Boot SE	95% CI	
			LLCI	ULCI
CS	−0.057	0.027	−0.107	−0.0003
SS	−0.069	0.032	−0.134	−0.011

Notes: CS, perceived certainty of sanctions; and SS, perceived severity of sanctions.

## Data Availability

The datasets used in this research are available upon request from the corresponding author. The data are not publicly available due to restrictions, i.e., privacy or ethical.
